# Supplementary material for: Pseudoscientific Health Beliefs and the Perceived Frequency of Causal Relationships
Source: Int J Environ Res Public Health. 2021 Oct 25;18(21):11196. doi: 10.3390/ijerph182111196 (PMC8583395; doi:10.3390/ijerph182111196)
Supplement: Supplementary file 1 [file ijerph-18-11196-s001.zip › ijerph-1405048-supplementary.pdf]

Supplementary Material

Supplementary file S1

Geographically-targeted postcodes

In order to capture greater variability in beliefs, we geo-targeted certain areas in the country that have low childhood-immunisation rates, and regional areas close to wind farms. The following postcodes were included in the geo-targeting of respondents, however there was little variability overall in the beliefs about Vaccination-Autism and wind turbine syndrome.

Low immunization regions	2000, 2481, 2482, 2134, 2095, 2478, 4560, 4567
Regions close to wind farms	2580, 2582, 2621, 3350, 3377, 5419, 5280, 5520

Supplementary file S2

Participant demographic

Table S1.

Absolute and percentage frequency of various demographic characteristics of survey respondents.

Demographics	Frequency	Percentage (%)	Cumulative Percentage (%)
Age group (years)			
18-24	32	15.2	15.2
25-40	74	35.2	50.5
41-64	75	35.7	86.2
>65	29	13.8	100
Rather not say	0	0	100
Gender			
Male	77	36.7	36.7
Female	133	63.3	100
Other	0	0	100
Rather not say	0	0	100
Parental status			
Guardian	112	53.3	53.3
Non-Guardian	98	46.7	100

Residential area			
Metropolitan	115	54.8	54.8
Regional	95	45.2	100
Highest educational level			
High school student	2	1.0	1.0
High school graduate or diploma equivalent	54	25.7	26.7
Trade, technical or vocational training	49	23.3	50.0
Associate degree	17	8.1	58.1
Undergraduate degree (e.g. Bachelor's Degree)	51	24.3	82.4
Masters degree	20	9.5	91.9
Doctorate	2	1.0	92.9
Professional degree (e.g. M.D., D.D.S., Ordination)	12	5.7	98.6
Other	3	1.4	100
Employment Status			
Student	15	7.14	7.14
Unemployed but looking for work	17	8.10	15.2
Unemployed and not looking for work	2	0.95	16.2
Unable to work	8	3.81	20.0
Military	0	0	20.0
Employed for wages	94	44.8	64.8
Self-employed	17	8.10	72.9
Home-maker	24	11.4	84.3
Retired	28	13.3	97.6
Other	5	2.4	100

Analysis S1

Contingency estimation and causal belief separated by order of question presentation

Although the order of question presentation (causal belief subcategory first or contingency estimation subcategory first) was counterbalanced between participants, it is possible that participants presented with the causal belief ratings first were primed to provide probability estimates that were consistent with their reported beliefs. To determine if the order of question presentation made a significant impact on the relationship between contingency estimates and causal ratings, we repeated the same hierarchical regression analysis for the two groups of participants separately. These results are reported in Table S2 and depicted in Figure S2. Overall there is no evidence that the order of question presentation produced a systematic difference in the strength of the correlation.

Table S2.

Summary statistics from the hierarchical regression with causal belief rating as the dependent variable, contingency estimates (perceived Δp) as the predictor variable, and mean-centred scores on the personality measures (Paranormal Belief Scale, Conscientiousness and Openness to Experience, and Locus of Control) as covariates (step one of hierarchical regression). The analysis is conducted separately as a function of the order of question presentation (Belief first vs Contingency first).

	Belief First	Contingency First
Herbal-Cold	$\Delta r^2 = .054$, $\Delta F(1,74) = 4.51$, $\beta = .235$, $p = .037$, $BF_{10} = 2.61$	$\Delta r^2 = .022$, $\Delta F(1,100) = 2.44$, $\beta = .158$, $p = .122$, $BF_{10} = 1.12$
Chiropractic-Backpain	$\Delta r^2 = .328$, $\Delta F(1,59) = 36.9$, $\beta = .604$, $p < .001$, $BF_{10} = 8.81e+4$	$\Delta r^2 = .303$, $\Delta F(1,84) = 39.7$, $\beta = .567$, $p < .001$, $BF_{10} = 4.86e+5$
Acupuncture-Pain	$\Delta r^2 = .110$, $\Delta F(1,46) = 6.77$, $\beta = 2.60$, $p = .012$, $BF_{10} = 5.63$	$\Delta r^2 = .105$, $\Delta F(1,65) = 9.45$, $\beta = .351$, $p = .003$, $BF_{10} = 16.1$
Vaccination-Autism	$\Delta r^2 = .007$, $\Delta F(1,56) = .585$, $\beta = .086$, $p = .448$, $BF_{10} = .465$	$\Delta r^2 = .001$, $\Delta F(1,71) = .088$, $\beta = .032$, $p = .768$, $BF_{10} = .357$

WIFI-Cancer	$\Delta r^2 < .001$, $\Delta F(1,66) = .015$, $\beta = .015, p = .902$, $BF_{10} = .458$	$\Delta r^2 < .001$, $\Delta F(1,93) = .001$, $\beta = .003, p = .974$, $BF_{10} = .428$
Smoking-Breathing problems	$\Delta r^2 = .177$, $\Delta F(1,81) = 18.6$, $\beta = .430, p < .001$, $BF_{10} = 453.1$	$\Delta r^2 = .077$, $\Delta F(1,106) = 9.60$, $\beta = .280, p = .002$, $BF_{10} = 19.3$
Exercise-Lifespan	$\Delta r^2 = .147$, $\Delta F(1,67) = 13.8$, $\beta = .397, p < .001$, $BF_{10} = 77.8$	$\Delta r^2 = .209$, $\Delta F(1,85) = 25.5$, $\beta = .470, p < .001$, $BF_{10} = 5.75e+3$

Analysis S2

Contingency estimation and proximity on causal belief

In order to determine if proximity to the someone who uses the putative cause *or* has experienced the outcome influences the extent to which people rely on the perceived contingency between events to form causal judgements, we classified participants into Low and High proximity based on the self-reported closest relationship selected on the proximity question. Proximity was presented as five multiple-choice options in decreasing levels of proximity (1 being most proximal and 5 being least proximal). An example of the proximity question can be found in Materials S4 below. A summary of the frequency of proximity to the putative cause or the outcome is presented in Table S3.

Table S3

Summary statistics of the closest relationship participants have to someone exposed to the putative cause (an index of proximity) in each target topic. Ratings were made by selecting one of five proximity options.

Proximity to	Frequency	Percentage (%)	Cumulative Percentage (%)
Someone with autism			
1 I have	6	2.9	2.9
2 I am very close to	38	18.1	21.0

3 I am somewhat close to	20	9.5	30.5
4 I am a little close to	36	17.1	47.6
5 I am not at all close to	110	52.4	100
Someone who takes herbal remedies			
1 I take	59	28.1	28.1
2 I am very close to	25	11.9	40.0
3 I am somewhat close to	21	10.0	50.0
4 I am a little close to	24	11.4	61.4
5 I am not at all close to	81	38.6	100
Someone who regularly visits a chiropractor			
1 I visit	36	17.1	17.1
2 I am very close to	38	18.2	35.2
3 I am somewhat close to	25	11.9	47.1
4 I am a little close to	32	15.2	62.4
5 I am not at all close to	79	37.6	100
Someone who regularly visits an acupuncturist			
1 I visit	23	10.9	10.9
2 I am very close to	18	8.6	19.5
3 I am somewhat close to	18	8.6	28.1
4 I am a little close to	20	9.5	37.6
5 I am not at all close to	131	62.4	100
Someone who lives close to or works on a wind farm			
1 I live close to or work on	7	3.3	3.3
2 I am very close to	7	3.3	6.7
3 I am somewhat close to	7	3.3	10.0

4 I am a little close to	13	6.2	16.2
5 I am not at all close to	176	83.8	100
Someone who regularly uses mobile phones and/or Wi-Fi			
1 I use	182	86.7	86.7
2 I am very close to	16	7.6	94.3
3 I am somewhat close to	8	3.8	98.1
4 I am a little close to	3	1.4	99.5
5 I am not at all close to	1	0.5	100
Someone who is or used to be a habitual smoker			
1 I am or used to be	76	36.2	36.2
2 I am very close to	59	28.1	64.3
3 I am somewhat close to	24	11.4	75.7
4 I am a little close to	17	8.1	83.8
5 I am not at all close to	34	16.2	100
Someone who is over the age of 65 and exercises regularly			
1 I am	14	6.7	6.7
2 I am very close to	75	35.7	42.4
3 I am somewhat close to	31	14.8	57.1
4 I am a little close to	23	11.0	68.1
5 I am not at all close to	67	31.9	100

We then classified participants as either Low or High in proximity: participants were classified as High in proximity if they selected option 1 (e.g. I visit a chiropractor) or 2 (e.g. I am very close to someone who visits a chiropractor), and Low in proximity if they selected options 3-5. Figure S1 depicts participants who were classified as High in proximity on the

left half of the figure, and Low in proximity on the right half of the figure, separated by targeted health beliefs (or topics). The frequency of High and Low proximity respondents in each topic area is denoted as a percentage of the total number of responses (N = 210).

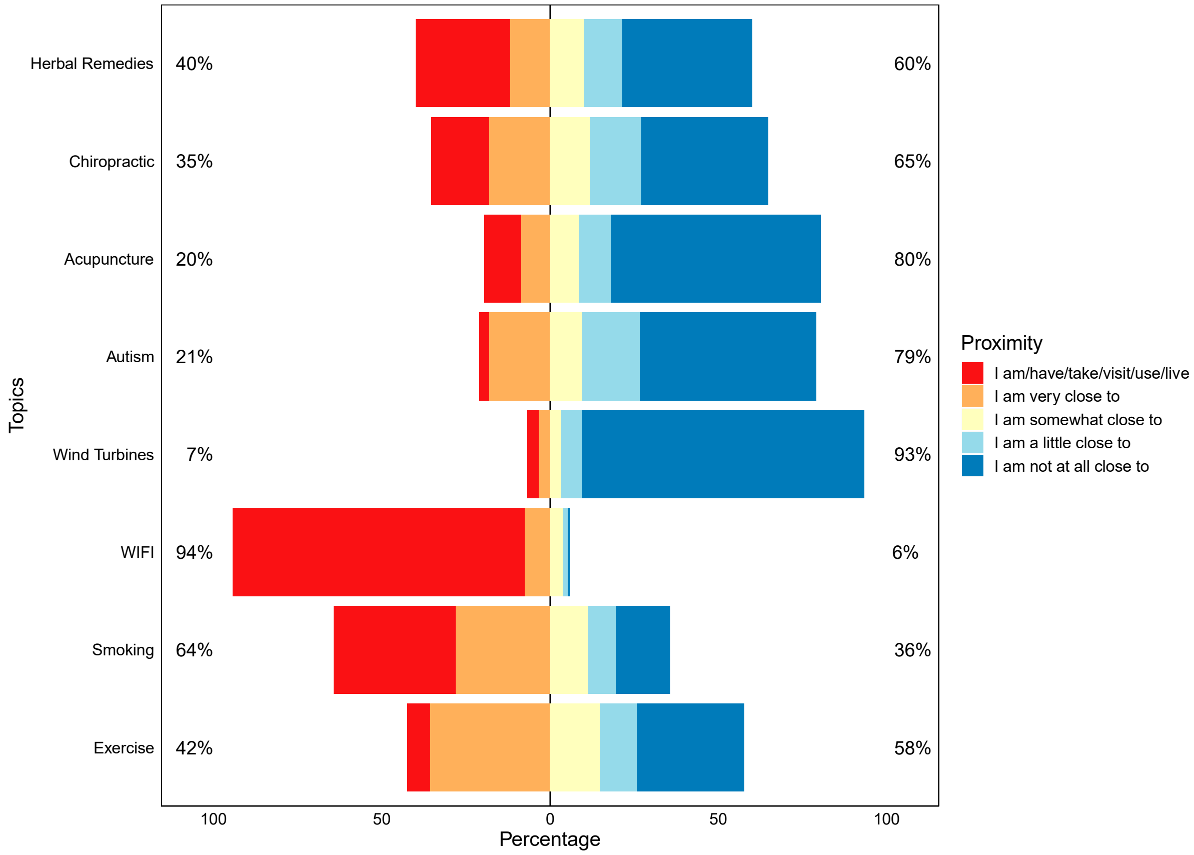

Figure S3. Frequency of respondent's proximity to the cue or outcome by topic area. Respondents (N = 210) selected their closest relationship to the cue or outcome by selecting one of five available choices ranging from 1 (high proximity/personal experience) to 5 (very low proximity). Plots on the left half of the plot area denotes proportion of selections that are high in proximity (choices 1 and 2), whereas the left half of the plot area denotes proportion of selections that are low in proximity (choices 3-5).

In order to determine the role of proximity in influencing the relationship between contingency estimation and causal belief, we first dummy coded proximity as 0 (Low proximity) and 1 (High proximity) and computed an interaction term for Δp and proximity and included this new interaction term as a predictor variable in the hierarchical regression together with the dummy coded proximity measure and raw Δp scores in a single step. This

allowed us to determine if there was a difference in the relationship between contingency estimates and causal belief as a function of low or high proximity to the putative cause or outcome. For this analysis, reported Bayes factor is the likelihood ratio of the alternative model which only included the interaction term, relative to the null model consisting of the four personality measures, and proximity and contingency estimates separately. These results are presented in Table S4. On the whole, our findings suggest that the relationship between perceived contingency and causal belief was not influenced by how proximal they were to someone who engages in the health behaviour or to the outcome. Where there was a significant change in the strength of the relationship between contingency estimates and causal ratings as a function of low and high proximity (acupuncture, exercise), the effect was in opposite directions.

Table S4.

Summary statistics of the role of proximity in influencing the relationship between perceived contingency and causal belief. Positive β values suggest an increase in the strength of the relationship between perceived contingency and causal belief when respondents were high in proximity than when they were low in proximity.

	Contingency Estimates x Proximity on Causal belief ratings
Herbal-Cold	$\beta = .054$, $t(1) = .629$<br>$p = .530$, $BF_{10} = .356$
Chiropractic-Backpain	$\beta = -.080$ $t(1) = -.821$<br>$p = .413$, $BF_{10} = .347$
Acupuncture-Pain	$\beta = .246$, $t(1) = 2.21$*<br>$p = .029$, $BF_{10} = 2.80$
Vaccination-Autism	$\beta = -.108$, $t(1) = -.907$<br>$p = .366$, $BF_{10} = .529$
Wind turbine syndrome	*NA*
WIFI-Cancer	$\beta = .033$, $t(1) = .085$<br>$p = .932$, $BF_{10} = .400$

Smoking-Breathing problems	$\beta = .151$, $t(1) = 1.23$<br>$p = .220$, $BF_{10} = .631$
Exercise-Lifespan	$\beta = -.226$, $t(1) = -2.21$*<br>$p = .029$, $BF_{10} = 2.50$

Participant's mean-centered scores on the Revised Paranormal Belief Scale, measures of Conscientiousness and Openess to Experience from the International Personality Pool Item (IPIP), and Levenson's Multidimensional Locus of Control Scale were entered as covariates in a linear regression analysis, and included in the null model in a Bayesian linear regression analysis.
*$p < .05$
** $p < .05$ *and* $BF_{10} > 3$

Analysis S3

Mean scores on individual differences measures

Average scores on each of the four personality measures (revised paranormal belief scale, conscientiousness scale, openness to experience scale, and Levenson's multidimensional locus of control scale) is presented in Figure S4.

Revised Paranormal Belief Scale. Belief in the paranormal was measured with the 26-item Revised Paranormal Belief Scale (rPBS; Tobayck & Milford, 1983). Each item was rated on a 7-point Likert scale; higher scores on the rPBS suggest stronger beliefs in the paranormal. Total score on the seven subscales (Traditional Religious Belief, Psi, Witchcraft, Superstition, Spiritualism, Extraordinary Life Forms, Precognition) were averaged to form a global mean paranormal belief score (*M* = 3.15, *SD* = 1.34).

Conscientiousness and openness to experience. The personality constructs Conscientiousness and Openness to Experience were measured concurrently on the 10-item International Personality Item Pool (IPIP) scale (Goldberg, 2006). The items were rated on a 5-point Likert scale from 1 (Very Inaccurate) to 5 (Very Accurate). The 10-item IPIP Scale has previously been validated against the NEO-PI-R (Costa & McCrae, 1992), Conscientiousness (*r* = .79) and Openness to Experience (*r* = .79), and is found to be an adequate and convenient tool for measuring personality factors (Donnellan, Oswald, Baird &

Lucas, 2006). Mean scores on the Conscientiousness scale was 2.97 (*SD* = 0.62), and 3.00 (*SD* = 0.59) on the Openness to Experience scale.

Levenson Multidimensional Locus of Control Scale. Participants completed the Levenson Multidimensional Locus of Control Scale (Levenson, 1973 & 1974) consisting of 24 items across three subscales: Internal Locus of Control, Powerful Others and Chance (the latter two subscales form the External Locus of Control Scale). Responses were made on a 6-point Likert scale from -3 (Strongly disagree) to +3 (Strongly agree). The total score on each individual subscale was calculated according to the standard Levenson procedure (+24 to total score of each subscale). We then averaged scores on all three subscales to form a global Locus of Control score (*M* = 53.9, *SD* = 4.00).

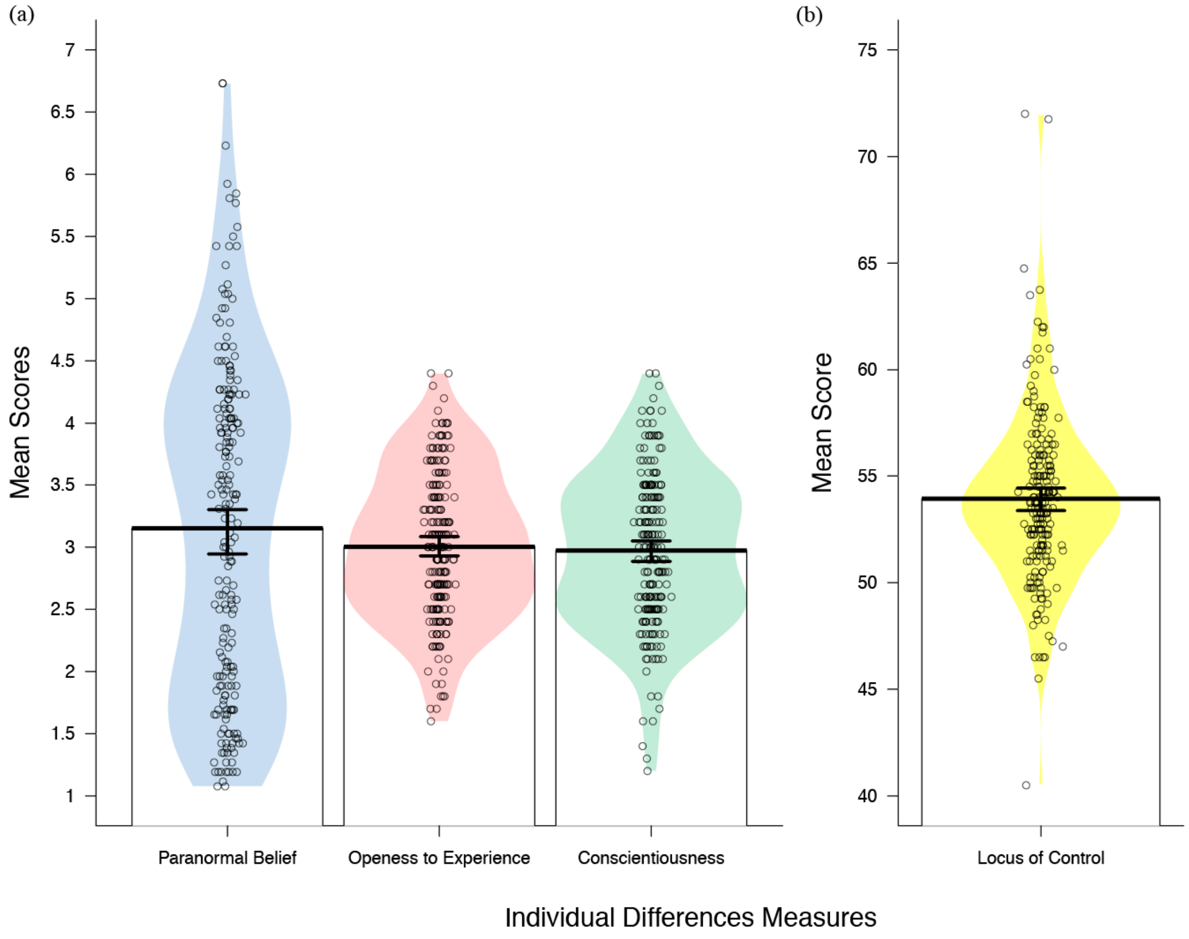

Figure S4. Mean scores(±SE) on the (a) Revised Paranormal Belief Scale, as well as Openness to Experience and Conscientiousness measured with the 10-item International Personality Item Pool (IPIP) scale, and (b) Levenson Multidimensional Locus of Control

scale. Each open circle represents an individual participant's score (N = 210 for each measure), and the coloured region depicts the density of scores along the Y-axis.

Supplementary Materials S4
Survey – "Contemporary Opinions on Factors that Impact Human Health"

Q111 You are invited to participate in a survey looking into the prevalence of people's beliefs in various environmental factors and therapies that may have positive or negative effects on health. There are no right or wrong answers. This is a sample of your own beliefs. You will also be required to complete two short personality questionnaires and various demographic questions at the end of the survey. Submitting your completed questionnaire is an indication of your consent to participate in the study. If you decide to take part in the study and then change your mind later, you are free to withdraw at any time. You can do this by exiting the survey. When you are ready to begin, click next at the bottom right corner of the screen.

Q113 Reminder: You are required to provide a response to every question. If your intended response is zero on a sliding scale, please click on the scale or move it along the axis before returning to zero to ensure your response has been recorded. If you do not move the scale at all, you will get an error message.

Q1.1 Childhood vaccination and autism
Belief: Vaccination during childhood is linked to subsequent development of autism

Q1.2 To what extent do you agree that childhood vaccination causes autism?

Strongly disagree | Somewhat disagree | Neither agree nor disagree | Somewhat agree | Strongly agree
0 10 20 30 40 50 60 70 80 90 100

Q1.4 To what extent would you recommend vaccinating young children?

Strongly against | Somewhat against | Undecided | Somewhat in favour | Strongly in favour
0 10 20 30 40 50 60 70 80 90 100

Q1.5 How much does it matter to you to know whether or not childhood vaccination causes autism?

Does not matter | Somewhat matters | Matters | Matters quite a lot | Matters very much
0 10 20 30 40 50 60 70 80 90 100

Q1.6 Select the closest relationship you have to someone who has autism.
- ❍ I have autism (1)
- ❍ I am very close to someone who has autism (2)
- ❍ I am somewhat close to someone who has autism (3)
- ❍ I am a little close to someone who has autism (4)
- ❍ I am not close at all to anyone who has autism (5)

Q1.7 Have you ever been vaccinated?

❍ Yes (1)

❍ No (2)

Q1.8 Of all the people you know, what percent of people have been vaccinated?

0 10 20 30 40 50 60 70 80 90 100

Percentage

Q1.9 Of those people who have been vaccinated, what proportion of them have autism? Percentage scale takes into account actual prevalence of autism in the population.

0 0.5 1 1.5 2 2.5 3 3.5 4 4.5 5

Percentage

Q1.10 Of all the people you know who have not been vaccinated, what proportion of them have autism? Percentage scale takes into account actual prevalence of autism in the population.

0 0.5 1 1.5 2 2.5 3 3.5 4 4.5 5

Percentage

Q2.1 Herbalism

Belief: The use of natural remedies (for example Zinc or Echinacea) are effective in treating the common cold

Q2.2 To what extent do you agree that herbal remedies are effective in treating the common cold?

Strongly disagree	Somewhat disagree	Neither agree nor disagree	Somewhat agree	Strongly agree

0 10 20 30 40 50 60 70 80 90 100

Q2.3 To what extent would you recommend using herbal remedies to treat the common cold?

Strongly against	Somewhat against	Undecided	Somewhat in favour	Strongly in favour

0 10 20 30 40 50 60 70 80 90 100

Q2.4 How much does it matter to you to know whether or not herbal remedies can treat the common cold?

Does not matter		Somewhat matters			Matters		Matters quite a lot			Matters very much
0	10	20	30	40	50	60	70	80	90	100

Q2.5 Select the closest relationship you have to someone who takes herbal remedies.
- ❍ I take herbal remedies (1)
- ❍ I am very close to someone who takes herbal remedies (2)
- ❍ I am somewhat close to someone who takes herbal remedies (3)
- ❍ I am a little close to someone who takes herbal remedies (4)
- ❍ I am not close at all to anyone who takes herbal remedies (5)

Q2.6 Have you ever taken herbal remedies to treat a common cold?
- ❑ Yes (1)
- ❑ No (2)

Display This Question:
If Have you ever taken herbal remedies to treat a common cold? Yes Is Selected

Q2.7 When you take herbal remedies, what is the severity of your cold before herbal treatment?

0 10 20 30 40 50 60 70 80 90 100

Percentage

Display This Question:
If Have you ever taken herbal remedies to treat a common cold? Yes Is Selected

Q2.8 When you take herbal remedies, what is the severity of your cold after herbal treatment?

0 10 20 30 40 50 60 70 80 90 100

Percentage

Q2.9 Of all the people you know, what percentage of people take herbal remedies to treat common cold?

0 10 20 30 40 50 60 70 80 90 100

Percentage

Display This Question:

If Of all the people you know, what percentage of people take herbal remedies to treat common cold? Percentage Is Greater Than 0

Q2.10 Of those people you know who have the common cold and take herbal remedies, what percentage have experienced an improvement in their condition?

0	10	20	30	40	50	60	70	80	90	100

Percentage

Q2.11 Of those people you know who have the common cold but do not take herbal remedies, what proportion have experienced an improvement in their condition?

0	10	20	30	40	50	60	70	80	90	100

Percentage

Q3.1 Chiropractic therapy is an effective treatment for back pain
Belief: The belief that disease results from a lack of normal nerve function, and manipulation of the spinal column and nervous system is effective for the treatment of back pain.

Q3.2 To what extent do you agree that chiropractic therapy is an effective treatment for back pain?

Strongly disagree	Somewhat disagree	Neither agree nor disagree	Somewhat agree	Strongly agree

0	10	20	30	40	50	60	70	80	90	100

Q3.3 To what extent would you recommend using chiropractic treatment for back pain?

Strongly against	Somewhat against	Undecided	Somewhat in favour	Strongly in favour

0	10	20	30	40	50	60	70	80	90	100

Q3.4 How much does it matter to you that chiropractic therapy can treat back pain?

Does not matter	Somewhat matters	Matters	Matters quite a lot	Matters very much

0	10	20	30	40	50	60	70	80	90	100

Q3.5 Select the closest relationship you have to someone who regularly visits a chiropractor.
- ❍ I visit a chiropractor (1)
- ❍ I am very close to someone who visits a chiropractor (2)
- ❍ I am somewhat close to someone who visits a chiropractor (3)
- ❍ I am a little close to someone who visits a chiropractor (4)
- ❍ I am not close at all to anyone who visits a chiropractor (5)

Q3.6 Have you ever visited a chiropractor for back pain?
- ❍ Yes (1)
- ❍ No (2)

Display This Question:
If Have you ever visited a chiropractor for back pain? Yes Is Selected

Q3.7 When you do visit a chiropractor for back pain, what is the severity of your back pain before chiropractic treatment?

0 10 20 30 40 50 60 70 80 90 100

Percentage

Display This Question:
If Have you ever visited a chiropractor for back pain? Yes Is Selected

Q3.8 When you do visit a chiropractor for back pain, what is the severity of your back pain after chiropractic treatment?

0 10 20 30 40 50 60 70 80 90 100

Percentage

Q3.9 Of all the people you know, what percentage of people visit a chiropractor for back pain?

0 10 20 30 40 50 60 70 80 90 100

Percentage

Display This Question:

If Of all the people you know, what percentage of people visit a chiropractor for back pain? Percentage Is Greater Than 0

Q3.10 Of those people you know who suffer from back pain and visit a chiropractor, what percentage have experienced an improvement in their condition?

0 10 20 30 40 50 60 70 80 90 100

Percentage

Display This Question:

If Of all the people you know, what percentage of people visit a chiropractor for back pain? Percentage Is Less Than 100

Q3.11 Of those people you know who suffer from back pain but do not visit a chiropractor, what percentage have experienced an improvement in their condition?

0 10 20 30 40 50 60 70 80 90 100

Percentage

Q34 Acupuncture is an effective treatment for pain

Belief: The insertion of fine needles into the body along the energy lines / meridians is an effective treatment for pain (e.g. back pain, neck pain, headaches). Please note that this is separate from a method of physiotherapy that uses needling to release tension from muscles.

Q35 To what extent do you agree that acupuncture is an effective treatment for pain?

Strongly disagree | Somewhat disagree | Neither agree nor disagree | Somewhat agree | Strongly agree

0 10 20 30 40 50 60 70 80 90 100

Q36 To what extent would you recommend using acupuncture to treat pain?

Strongly against | Somewhat against | Undecided | Somewhat in favour | Strongly in favour

0 10 20 30 40 50 60 70 80 90 100

Q37 How much does it matter to you if acupuncture can treat pain?

Does not matter | Somewhat matters | Matters | Matters quite a lot | Matters very much

0 10 20 30 40 50 60 70 80 90 100

Q38 How close are you to someone who visits an acupuncturist?
- ❍ I visit an acupuncturist (1)
- ❍ I am very close to someone who visits an acupuncturist (2)
- ❍ I am somewhat close to someone who visits an acupuncturist (3)
- ❍ I am a little close to someone who visits an acupuncturist (4)
- ❍ I am not close at all to anyone who visits an acupuncturist (5)

Q40 Have you ever visited an acupuncturist for pain?
- ❍ Yes (1)
- ❍ No (2)

Display This Question:
If Have you ever visited an acupuncturist for pain? Yes Is Selected

Q41 When you do visit an acupuncturist for pain, what is the severity of your pain before acupuncture?

0 10 20 30 40 50 60 70 80 90 100

Percentage

Q42 When you do visit an acupuncturist for pain, what is the severity of your pain after acupuncture?

0 10 20 30 40 50 60 70 80 90 100

Percentage

Q43 Of all the people you know, what percentage of people visit an acupuncturist for pain?

0 10 20 30 40 50 60 70 80 90 100

Percentage

Condition: Percentage Is Less Than or Equal to 0. Skip To: Of all the people you know who do not....

Q44 Of all the people you know who suffer from pain and visit an acupuncturist, what proportion have experienced an improvement in their condition?

0 10 20 30 40 50 60 70 80 90 100

Percentage

Q45 Of all the people you know who suffer from pain but do not visit an acupuncturist, what proportion have experienced an improvement in their condition?

0 10 20 30 40 50 60 70 80 90 100

Percentage

Q46 Wind turbine syndrome

Belief: Exposure to wind turbines can cause adverse health effects including memory loss, disturbed sleep and headaches (even when the turbine cannot be heard).

Q46 To what extent do you agree that wind turbines cause adverse health effects?

Strongly disagree		Somewhat disagree			Neither agree nor disagree		Somewhat agree			Strongly agree
0	10	20	30	40	50	60	70	80	90	100

Q47 To what extent would you recommend the development of wind farms in Australia?

Strongly against		Somewhat against			Undecided		Somewhat in favour			Strongly in favour
0	10	20	30	40	50	60	70	80	90	100

Q48 How much does it matter to you if wind turbines cause adverse health effects?

Does not matter		Somewhat matters			Matters		Matters quite a lot			Matters very much
0	10	20	30	40	50	60	70	80	90	100

Q49 Select the closest relationship you have to someone who lives close to or works on a wind farm.

- ❍ I live close to or work on a wind farm (1)
- ❍ I am very close to someone who lives close to or works on a wind farm (2)
- ❍ I am somewhat close to someone who lives close to or works on a wind farm (3)
- ❍ I am a little close to someone who lives close to or works on a wind farm (4)
- ❍ I am not at all close to anyone who lives close to or works on a wind farm (5)

Q52 Have you ever been exposed to wind turbines?

- ❍ Yes (1)
- ❍ No (2)

Display This Question:
If Have you ever been exposed to wind turbines? Yes Is Selected

Q50 Have you ever experience adverse health effects (e.g. headaches) after exposure to wind turbines?
- ❍ Yes (1)
- ❍ No (2)

Display This Question:
If Have you ever experience adverse health effects (e.g. headaches) after exposure to wind turbines? Yes Is Selected

Q51 When you are around wind turbines, how frequently do you experience these negative health effects (e.g. memory loss, disturbed sleep, headaches)?

Never		Rarely			Occasionally		Frequently			Always
0	10	20	30	40	50	60	70	80	90	100

Q53 When you are not around wind turbines, how frequently do you experience these negative health effects (e.g. memory loss, disturbed sleep, headaches)?

Never		Rarely			Occasionally		Frequently			Always
0	10	20	30	40	50	60	70	80	90	100

Q54 Of all the people you know, what percentage of them live close to or work on a wind farm?

0	10	20	30	40	50	60	70	80	90	100

Percentage

Display This Question:
If Of all the people you know, what percentage of them live close to or work on a wind farm? Percentage Is Greater Than 0

Q55 Of all the people you know who live close to or work on a wind farm, what proportion have experienced adverse health effects (e.g. memory loss, disturbed sleep, headaches)?

0	10	20	30	40	50	60	70	80	90	100

Percentage

Display This Question:
If Of all the people you know, what percentage of them live close to or work on a wind farm? Percentage Is Greater Than 0

Q56 Of all the people you know who live close to or work on a wind farm, what proportion have not experienced adverse health effects (e.g. memory loss, disturbed sleep, headaches)?

0 10 20 30 40 50 60 70 80 90 100

Percentage

Q57 ***Belief: Radiation from mobile phones and Wi-Fi causes cancer***

Q58 To what extent do you agree that radiation from mobile phones and Wi-Fi causes cancer?

Strongly disagree		Somewhat disagree			Neither agree nor disagree		Somewhat agree			Strongly agree
0	10	20	30	40	50	60	70	80	90	100

Q59 To what extent would you recommend policies that restrict the radiation released from mobile phones and Wi-Fi?

Strongly against		Somewhat against			Undecided		Somewhat in favour			Strongly in favour
0	10	20	30	40	50	60	70	80	90	100

Q60 How much does it matter to you if mobile phone and Wi-Fi radiation causes adverse health effects such as cancer?

Does not matter		Somewhat matters			Matters		Matters quite a lot			Matters very much
0	10	20	30	40	50	60	70	80	90	100

Q61 Select the closest relationship you have to someone who regularly uses mobile phones and/or Wi-Fi.

- ❍ I regularly use mobile phones and/or Wi-Fi (1)
- ❍ I am very close to someone who regularly uses mobile phones and/or Wi-Fi (2)
- ❍ I am somewhat close to someone who regularly uses mobile phones and/or Wi-Fi (3)
- ❍ I am a little close to someone who regularly uses mobile phones and/or Wi-Fi (4)
- ❍ I am not close at all to anyone who regularly uses mobile phones and/or Wi-Fi (5)

Q62 Do you use mobile phones and/or Wi-Fi regularly?

- ❍ Yes (1)
- ❍ No (2)

Q63 Of all the people you know, what percentage of people regularly use mobile phones and/or Wi-Fi?

0 10 20 30 40 50 60 70 80 90 100

Percentage

Display This Question:

If Of all the people you know, what percentage of people regularly use mobile phones and/or Wi-Fi? Percentage Is Greater Than 0

Q64 Of those people between the age of 60-80 who regularly use mobile phones and/or Wi-Fi, what percentage of them have been diagnosed with cancer? Percentage scale takes into account the actual prevalence of cancer in the population.

0 1 2 3 4 5 6 7 8 9 10

Percentage

Q65 Of all the people you know between the age of 60-80 who do not regularly use mobile phones and/or Wi-Fi, what percentage of them have cancer? Percentage scale takes into account the actual prevalence of cancer in the population.

0 1 2 3 4 5 6 7 8 9 10

Percentage

Q66 ***Belief: Prolonged smoking causes heart, lung and breathing problems***

Q67 To what extent do you agree that smoking causes heart, lung and breathing problems?

Strongly disagree | Somewhat disagree | Neither agree nor disagree | Somewhat agree | Strongly agree

0 10 20 30 40 50 60 70 80 90 100

Q68 To what extent would you recommend policies that limit the sale of cigarettes?

Strongly against | Somewhat against | Undecided | Somewhat in favour | Strongly in favour

0 10 20 30 40 50 60 70 80 90 100

Q69 How much does it matter to you if smoking causes heart, lung and breathing problems?

Does not matter		Somewhat matters			Matters		Matters quite a lot			Matters very much
0	10	20	30	40	50	60	70	80	90	100

Q70 Select the closest relationship you have to someone who is or used to be a habitual smoker.

- ❍ I am or used to be a habitual smoker (1)
- ❍ I am very close to someone who is or used to be a habitual smoker (2)
- ❍ I am somewhat close to someone who is or used to be a habitual smoker (3)
- ❍ I am a little close to someone who is or used to be a habitual smoker (4)
- ❍ I am not close at all to anyone who is or used to be a habitual smoker (5)

Q71 Do you currently or have you ever smoked regularly?

- ❍ Yes (1)
- ❍ No (2)

Display This Question:

If Do you currently or have you ever smoked regularly? Yes Is Selected

Q72 When you smoke regularly, how frequently do you experience heart, lung and breathing problems?

Never		Rarely			Occasionally		Frequently			Always
0	10	20	30	40	50	60	70	80	90	100

Q73 When you do not smoke regularly, how frequently do you experience heart, lung and breathing problems?

Never		Rarely			Occasionally		Frequently			Always
0	10	20	30	40	50	60	70	80	90	100

Q74 Of all the people you know, what percentage of people smoke regularly or have smoked regularly in the past?

0	10	20	30	40	50	60	70	80	90	100

Percentage

Display This Question:
If Of all the people you know, what percentage of people smoke regularly or have smoked regularly in the past? Percentage Is Greater Than 0

Q75 Of those people who smoke regularly or have smoked regularly in the past, what proportion of them have experienced heart, lung and breathing problems?

0 10 20 30 40 50 60 70 80 90 100

Percentage

Q76 Of all the people you know who do not smoke regularly, what proportion of them have experienced heart, lung, and breathing problems?

0 10 20 30 40 50 60 70 80 90 100

Percentage

Q78 ***Belief: Regular exercise increases lifespan***

Q79 To what extent do you agree that regular exercise increases lifespan?

Strongly disagree		Somewhat disagree			Neither agree nor disagree		Somewhat agree			Strongly agree
0	10	20	30	40	50	60	70	80	90	100

Q80 To what extent would you recommend policies that promote regular exercise in communities?

Strongly against		Somewhat against			Undecided		Somewhat in favour			Strongly in favour
0	10	20	30	40	50	60	70	80	90	100

Q81 How much does it matter to you if regular exercise increases lifespan?

Does not matter		Somewhat matters			Matters		Matters quite a lot			Matters very much
0	10	20	30	40	50	60	70	80	90	100

Q82 Select the closest relationship you have to someone over the age of 65 and exercises regularly.

- ❍ I am over the age of 65 and exercise regularly (1)
- ❍ I am very close to someone over the age of 65 and exercises regularly (2)
- ❍ I am somewhat close to someone over the age of 65 and exercises regularly (3)
- ❍ I am a little close to someone over the age of 65 and exercises regularly (4)
- ❍ I am not close at all to anyone over the age of 65 and exercises regularly (5)

Q83 Are you over the age of 65?

- ❍ Yes (1)
- ❍ No (2)

Q114 Do you exercise regularly?

- ❍ Yes (1)
- ❍ No (2)

Q115 Do you have very good health?

- ❍ Yes (1)
- ❍ No (2)

Q84 Of all the people you know over the age of 65, what percentage of them exercise regularly?

0 10 20 30 40 50 60 70 80 90 100

Percentage

Display This Question:
If Of all the people you know over the age of 65, what percentage of them exercise regularly? Percentage Is Greater Than 0

Q85 Of older people you know who exercised regularly, what percentage of them have had especially long lifespans?

0 10 20 30 40 50 60 70 80 90 100

Percentage

Q86 Of older people you know who have not exercised regularly, what percentage of them have had especially long lifespans?

0 10 20 30 40 50 60 70 80 90 100

Percentage

Q87 Restrictive diets
Belief: Certain foods can cause you to feel bad

Q88 Do you avoid certain foods for any particular health reason? We aren't asking if you try to eat healthy in general, but if you try to avoid a particular food because you feel that you are allergic or sensitive to it or it makes you feel bad. If you have multiple sensitivities / intolerances / allergies, please choose the one that is most problematic for you. Select N/A if you do not actively avoid any particular food

- ❍ Peanuts (1)
- ❍ Tree nuts (2)
- ❍ Dairy / Cow's milk (3)
- ❍ Eggs (4)
- ❍ Gluten / Wheat (5)
- ❍ Fish (6)
- ❍ Shellfish (7)
- ❍ Soy (8)
- ❍ Tyramine (9)
- ❍ Preservatives and Additives (10)
- ❍ FODMAP (11)
- ❍ Other (12) ____________________
- ❍ N/A I do not actively avoid any particular food (13)

Condition: N/A I do not actively avoid... Is Selected. Skip To: To what extent would....

Q89 What happens when you eat this kind of food? Check all that apply

- ❑ Stomach ache/Diarrhoea/Vomiting (1)
- ❑ Skin rashes/Hives/Eczema (2)
- ❑ Headaches (3)
- ❑ Blood in stool (4)
- ❑ Other (5) ____________________

Q90 How restrictive is your diet?

- ❍ I never eat this type of food (1)
- ❍ I usually eat this type of food at least once a year (2)
- ❍ I usually eat this type of food at least once a month (3)
- ❍ I usually eat this type of food at least once per week (4)
- ❍ I usually eat this type of food multiple times a week (5)
- ❍ I usually eat this food daily (6)

Q91 How much do you miss eating this type of food?

Not at all | A little | Moderately | A lot | Severely

0 10 20 30 40 50 60 70 80 90 100

Q92 How disruptive is your restrictive diet to your life?

Not at all		A little			Moderately		A lot		Severely	
0	10	20	30	40	50	60	70	80	90	100

Q93 To what extent do you agree that restricting your diet helps you to feel better?

Strongly disagree		Somewhat disagree			Neither agree nor disagree		Somewhat agree			Strongly agree
0	10	20	30	40	50	60	70	80	90	100

Q94 Have you ever had a medical professional recommend that you restrict your diet to avoid this type of food? If so, what sort of medical professional?

- ❍ Licensed medical doctor (1)
- ❍ Licensed dietician (2)
- ❍ Chiropractor (3)
- ❍ Alternative / complementary medicine practitioner or 'healer' (4)
- ❍ Other (5) ____________________
- ❍ No - I have never been recommended to avoid any particular food (6)

Q95 Have you ever had medical testing to diagnose your allergy, sensitivity, or intolerance?

- ❍ Yes (24)
- ❍ No (23)

Display This Question:

If Have you ever had medical testing to diagnose your allergy, sensitivity, or intolerance? No Is Selected

Q96 Please explain what sort of testing

Display This Question:

If Have you ever had medical testing to diagnose your allergy, sensitivity, or intolerance? No Is Selected

Q97 What was the outcome of the testing?

- ❍ I have an allergy (1)
- ❍ I have a sensitivity / intolerance (2)
- ❍ The testing was inconclusive (3)
- ❍ I do not have an allergy, sensitivity or intolerance (4)
- ❍ Not sure / I'm confused (5)
- ❍ Other (please explain) (6) ____________________

Q98 Have you ever tried to completely stop eating the food for a couple days or longer, and then started to eat it again in order to figure out if the food makes you feel bad? Sometimes this is called an 'elimination-challenge' diet.
- ❍ Yes (24)
- ❍ No (23)

Q99 To what extent would you recommend that people with habitual stomach aches or diarrhoea try a restrictive diet?

Strongly against		Somewhat against			Undecided		Somewhat in favour			Strongly in favour
0	10	20	30	40	50	60	70	80	90	100

Q100 How much does it matter to you if practicing a restrictive diet improves habitual stomach aches or diarrhoea?

Does not matter		Somewhat matters			Matters		Matters quite a lot			Matters very much
0	10	20	30	40	50	60	70	80	90	100

Q101 Select the closest relationship you have to someone who practices a restrictive diet.
- ❍ I practice a restrictive diet (1)
- ❍ I am close to someone who practices a restrictive diet (2)
- ❍ I am somewhat close to someone who practices a restrictive diet (3)
- ❍ I am a little close to someone who practices a restrictive diet (4)
- ❍ I am not close at all to anyone who practices a restrictive diet (5)

Q102 Of all the people you know, what percentage of people practice a restrictive diet to improve habitual stomach aches or diarrhoea?

0	10	20	30	40	50	60	70	80	90	100

Percentage

Display This Question:

If Of all the people you know, what percentage of people practice a restrictive diet to improve habitual stomach aches or diarrhoea? Percentage Is Greater Than 0

Q103 Of those people who practice a restrictive diet, what percentage of them frequently experience stomach aches or diarrhoea when adhering to the diet?

0	10	20	30	40	50	60	70	80	90	100

Percentage

Q104 Of all the people you know who do not practice a restrictive diet, what percentage of them frequently experience stomach aches or diarrhoea when they aren't adhering to the diet?

0 10 20 30 40 50 60 70 80 90 100

Percentage

Q112 You will now be asked to complete a series of short personality questionnaires. When you are ready to begin, click next.

Materials S5
Revised Paranormal Belief Scale

Q106 Please select a number next to each item to indicate how much you agree or disagree with that item. Use the numbers as indicated below. There are no right or wrong answers. This is a sample of your own beliefs and attitudes.

1=Strongly Disagree
2=Moderately Disagree
3=Slightly Disagree
4=Uncertain
5=Slightly Agree
6=Moderately Agree
7=Strongly Agree

	1 Strongly Disagree	2 Moderately Disagree	3 Slightly Disagree	4 Uncertain	5 Slightly Agree	6 Moderately Agree	7 Strongly Agree
1. The soul continues to exist though the body may die	❍	❍	❍	❍	❍	❍	❍
2. Some individuals are able to levitate (lift) objects through mental forces	❍	❍	❍	❍	❍	❍	❍
3. Black magic really exists	❍	❍	❍	❍	❍	❍	❍
4. Some omens, such as a black cat crossing, can bring bad luck	❍	❍	❍	❍	❍	❍	❍
5. Your mind or soul can leave your body and travel (astral projection)	❍	❍	❍	❍	❍	❍	❍
6. The abominable snowman of Tibet exists	❍	❍	❍	❍	❍	❍	❍
7. Astrology is a way to accurately predict the future							

❍ ❍ ❍ ❍ ❍ ❍ ❍

8. There is a devil

❍ ❍ ❍ ❍ ❍ ❍ ❍

9. Psychokinesis, the movement of objects through psychic powers, does exist

❍ ❍ ❍ ❍ ❍ ❍ ❍

10. Witches who have supernatural powers exist

❍ ❍ ❍ ❍ ❍ ❍ ❍

11. If you break a mirror, you will have bad luck

❍ ❍ ❍ ❍ ❍ ❍ ❍

12. During altered states, such as sleep or trances, the spirit can leave the body

❍ ❍ ❍ ❍ ❍ ❍ ❍

13. The Loch Ness monster of Scotland exists

❍ ❍ ❍ ❍ ❍ ❍ ❍

14. The horoscope accurately tells a person’s future

❍ ❍ ❍ ❍ ❍ ❍ ❍

15. I believe in God

❍ ❍ ❍ ❍ ❍ ❍ ❍

16. A person’s thoughts can influence the movement of a physical object

❍ ❍ ❍ ❍ ❍ ❍ ❍

17. Through the use of formulas and incantations, it is possible to cast spells on persons

❍ ❍ ❍ ❍ ❍ ❍ ❍

18. Some numbers, for example 13, are unlucky

❍ ❍ ❍ ❍ ❍ ❍ ❍

19. Reincarnation does occur

❍ ❍ ❍ ❍ ❍ ❍ ❍

20. There is life on other planets

❍ ❍ ❍ ❍ ❍ ❍ ❍

21. Some psychics can accurately predict the future

❍ ❍ ❍ ❍ ❍ ❍ ❍

22. There is a heaven and a hell

❍ ❍ ❍ ❍ ❍ ❍ ❍

23. Telepathic mind reading is not possible

❍ ❍ ❍ ❍ ❍ ❍ ❍

24. There are actual cases of witchcraft

❍ ❍ ❍ ❍ ❍ ❍ ❍

25. Dead people can send messages to their loved ones

❍ ❍ ❍ ❍ ❍ ❍ ❍

26. Some people have an unexplained ability to predict the future.

❍ ❍ ❍ ❍ ❍ ❍ ❍

Scoring
Note: Item 23 is reverse scored.
Average score over the 26 items were taken as a measure of paranormal belief. Subscales can be calculated as below:

Traditional Religious Belief = Mean of items (1,8,15,22)
Psi = Mean of items (2,9,16,23)
Witchcraft = Mean of items (3,10,17,24)
Superstition = Mean of items (4,11,18)
Spiritualism = Mean of items (5,12,19,25)
Extraordinary life forms = Mean of items (6,13,20)
Precognition = Mean of items (7,14,21,26)

Materials S6
Conscientiousness and Openness to Experience

Q107 Describe yourself as you generally are now, not as you wish to be in the future. Describe yourself as you honestly see yourself, in relation to other people you know of the same sex as you are, and roughly your same age. So that you can describe yourself in an honest manner, your responses will be kept in absolute confidence.
Indicate for each statement whether it is:

1= Very Inaccurate
2= Moderately Inaccurate
3= Neither Accurate nor Inaccurate
4= Moderately Accurate
5= Very Accurate

	1<br>Very Inaccurate	2<br>Moderately Inaccurate	3<br>Neither Accurate nor Inaccurate	4<br>Moderately Accurate	5<br>Very Accurate
1. I believe in the importance of art					
	❍	❍	❍	❍	❍
2. I waste my time					
	❍	❍	❍	❍	❍
3. I am not interested in abstract ideas					

❍ ❍ ❍ ❍ ❍

4. I am always prepared

❍ ❍ ❍ ❍ ❍

5. Have a vivid imagination

❍ ❍ ❍ ❍ ❍

6. I find it difficult to get down to work

❍ ❍ ❍ ❍ ❍

7. Do not like art

❍ ❍ ❍ ❍ ❍

8. I pay attention to details

❍ ❍ ❍ ❍ ❍

9. Tend to vote for progressive political candidates

❍ ❍ ❍ ❍ ❍

10. I do just enough work to get by

❍ ❍ ❍ ❍ ❍

11. Avoid philosophical discussions

❍ ❍ ❍ ❍ ❍

12. I get chores done right away

❍ ❍ ❍ ❍ ❍

13. Carry the conversation to a higher level

❍ ❍ ❍ ❍ ❍

14. I don’t see things through

❍ ❍ ❍ ❍ ❍

15. I do not enjoy going to art museums

❍ ❍ ❍ ❍ ❍

16. I carry out my plans

❍ ❍ ❍ ❍ ❍

17. Enjoy hearing new ideas

❍ ❍ ❍ ❍ ❍

18. I shirk my duties

❍ ❍ ❍ ❍ ❍

19. Tend to vote for conservative political candidates

❍ ❍ ❍ ❍ ❍

20. I make plans and stick to them

	❍	❍	❍	❍	❍

Scoring
Note: Items 2,3,6,7,10,11,14,15,18,19 are reverse-scored.
Average score over the 10 relevant items on each scale were taken as a measure of Conscientiousness and Openness to Experience.

Conscientiousness = Mean of items (2,4,6,8,10,12,14,16,18,20)
Openness to Experience = Mean of items (1,3,5,7,9,11,13,15,17,19)

Materials S7
Levenson Multidimensional Locus of Control Scale

Q108 For each of the following statements, indicate the extent to which you agree or disagree by writing in the appropriate number

-3 = Strongly disagree
-2 = Disagree somewhat
-1 = Slightly disagree
+1 = Slightly agree
+2 = Agree somewhat
+3 = Strongly agree

	-3 Strongly Disagree	-2 Disagree Somewhat	-1 Slightly Disagree	+1 Slightly Agree	+2 Agree Somewhat	+3 Strongly Agree
1. Whether or not I get to be a leader depends mostly on my ability	❍	❍	❍	❍	❍	❍
2. To a great extent my life is controlled by accidental happenings	❍	❍	❍	❍	❍	❍
3. I feel like what happens in my life is mostly determined by powerful people	❍	❍	❍	❍	❍	❍
4. Whether or not I get into a car accident depends mostly on how good a driver I am	❍	❍	❍	❍	❍	❍
5. When I make plans, I am almost certain to make them work	❍	❍	❍	❍	❍	❍
6. Often there is no chance of protecting my personal interests from bad luck	❍	❍	❍	❍	❍	❍
7. When I get what I want, it’s usually because I’m lucky	❍	❍	❍	❍	❍	❍

8. Although I might have good ability, I will not be given leadership responsibility without appealing to those in positions of power

❍ ❍ ❍ ❍ ❍ ❍

9. How many friends I have depends on how nice a person I am

❍ ❍ ❍ ❍ ❍ ❍

10. I have often found that what is going to happen will happen

❍ ❍ ❍ ❍ ❍ ❍

11. My life is chiefly controlled by powerful others

❍ ❍ ❍ ❍ ❍ ❍

12. Whether or not I get into a car accident is mostly a matter of luck

❍ ❍ ❍ ❍ ❍ ❍

13. People like myself have very little chance of protecting our personal interests when they conflict with those of strong pressure groups

❍ ❍ ❍ ❍ ❍ ❍

14. It's not always wise for me to plan too far ahead because many things turn out to be a matter of good or bad fortune

❍ ❍ ❍ ❍ ❍ ❍

15. Getting what I want requires pleasing those people above me

❍ ❍ ❍ ❍ ❍ ❍

16. Whether or not I get to be a leader depends on whether I'm lucky enough to be in the right place at the right time

❍ ❍ ❍ ❍ ❍ ❍

17. If important people were to decide they didn't like me, I probably wouldn't make any friends

❍ ❍ ❍ ❍ ❍ ❍

18. I can pretty much determine what will happen in my life

❍ ❍ ❍ ❍ ❍ ❍

19. I am usually able to protect my personal interests

❍ ❍ ❍ ❍ ❍ ❍

20. Whether or not I get into a car accident depends mostly on the other driver

❍ ❍ ❍ ❍ ❍ ❍

21. When I get what I want, it's usually because I worked hard for it

❍ ❍ ❍ ❍ ❍ ❍

22. My life is determined by my own actions

❍ ❍ ❍ ❍ ❍ ❍

23. It's chiefly a matter of fate whether or not I have a few friends or many friends

❍ ❍ ❍ ❍ ❍ ❍

Scoring

Total responses for the items listed for each of the three parts of the scale; add +24 to each of the three totals (Levenson, 1973). Average score on all three subscales were taken as measure of locus of control.

Internal Locus of Control: Total your responses for items 1,4,5,9,18,19,21,23

Powerful Others: Total your responses for items 3,8,11,13,15,17,20,22

Chance: Total your responses for items 2,6,7,10,12,14,16,24
